# Point-of-care ultrasound induced changes in management of unselected patients in the emergency department - a prospective single-blinded observational trial

**DOI:** 10.1186/s13049-020-00740-x

**Published:** 2020-05-29

**Authors:** Jesper Weile, Christian A. Frederiksen, Christian B. Laursen, Ole Graumann, Erik Sloth, Hans Kirkegaard

**Affiliations:** 1 Emergency Department, Regional Hospital Herning, Herning, Denmark; 2 Research Center for Emergency Medicine, Aarhus University Hospital, Palle Juul-Jensens Blvd. 161 (J 103), 8200 Aarhus, Denmark; 3 Department of Cardiology, Aarhus University Hospital, Aarhus, Denmark; 4 Department of Respiratory Medicine, Odense University Hospital, Odense, Denmark; 5 Department of Radiology, Odense University Hospital, Odense, Denmark; 6 Institute of Clinical Research, University of Southern Denmark, Odense, Denmark; 7 University of Cape Town, Cape Town, South Africa; 8 Department of Clinical Medicine, Aarhus University, Aarhus, Denmark

## Abstract

**Background:**

Point-of-Care ultrasound (POCUS) changes the management in specific groups of patients in the Emergency Department (ED). It seems intuitive that POCUS holds an unexploited potential on a wide variety of patients. However, little is known about the effect of ultrasound on the broad spectrum of unselected patients in the ED. This study aimed to identify the effect on the clinical management if POCUS was applied on unselected patients. Secondarily the study aimed to identify predictors of ultrasound changing management.

**Methods:**

This study was a blinded observational single center trial. A basic whole body POCUS protocol was performed in extension to the physical examination. The blinded treating physicians were interviewed about the presumptive diagnosis and plan for the patient. Subsequently the physicians were unblinded to the POCUS results and asked to choose between five options regarding the benefit from POCUS results.

**Results:**

A total of 403 patients were enrolled in this study. The treating physicians regarded POCUS examinations influence on the diagnostic workup or treatment as following: 1) No new information: 249 (61.8%), 2) No further action: 45 (11.2%), 3) Further diagnostic workup needed: 52 (12.9%), 4) Presumptive diagnosis confirmed 38 (9.4%), and 5) Immediate treatment needed: 19 (4.7%). Predictors of beneficial ultrasound were: (a) triage > 1, (b) patient comorbidities (cardiac disease, hypertension or lung disease), or (c) patients presenting with abdominal pain, dyspnea, or syncope.

**Conclusion:**

POCUS was found to be potentially beneficial in 27.0% of all patients. High triage score, known cardiac disease, hypertension, pulmonary diseases, a clinical presentation with abdominal pain, dyspnea, or syncope are predictors of this. Future research should focus on patient-important outcomes when applying POCUS on these patients.

**Trial registration:**

The trail was registered prior to patient inclusion with the Danish Data Protection Agency (https://www.datatilsynet.dk/ Case no: 1–16–02-603-14) and Clinical Trials (www.clinicaltrials.gov/ Protocol ID: DNVK1305018).

## Background

Ultrasound has been applied widely since the 1950’ies without any deleterious effects or discomfort to the patients [1, 2]. Point-of-care ultrasound (POCUS) refers to simple ultrasonographic examinations performed at the bedside answering dichotomous (yes/no) clinical questions [3, 4]. Diagnostic accuracy increases and patient management changes if POCUS is performed in critical patients [5–8]. However, POCUS is only applied on a small percentage of patients presenting to the ED [9–11].

Previous research has focused on selected groups of patients, such as trauma, shock, dyspnea, or critically ill patients, or patients with an already known diagnosis. Furthermore, previous studies have been designed for specific evaluations such as the heart or the lungs [5, 12–18]. These patients only represent a small sample of the patients seen in the ED [19–21].

Performing POCUS has been advocated as an amendment to the physical examination [22]. The physical exam is applicable as a screening tool to all patients visiting the ED [23], however some patients are bound to have higher benefit from POCUS than others. No previous studies have investigated the impact of POCUS on unselected patients in a broad population in an ED. To investigate the usefulness of POCUS on a broad population it is necessary to clarify the usefulness of positive findings to the treating physicians. When this knowledge is obtained it can be investigated if the impact is higher on certain patient subgroups.

The purpose of this study was to investigate the changes in patient management induced by POCUS as an amendment to the physical examination on all patients in the ED. Secondarily the study aimed to investigate if triage level, specific known diseases, or specific clinical presentations were predictors of physician-perceived relevance of whole-body ultrasound.

## Methods

This explorative study was a prospective blinded observational single center trial in a mixed urban/rural ED in Denmark with an annual uptake of approximately 35,000 patients. The department only received patients who were referred from either general practitioner or by ambulance following emergency calls. Some patients bypassed the ED: Patients with suspected acute myocardial infarction were transferred directly to a catheterization laboratory. Stroke patients who were candidates for thrombolysis or mechanical thrombectomy, women in labor, or children with a presumed medical emergency also bypassed the ED.

### Study outline

The patients were approached for inclusion in the study after receiving standard care with initial triage and primary assessment including physical examination. Within 2 h after the initial physical examination the principal investigator completed a whole-body point-of-care examination including focused cardiac ultrasound, focused lung ultrasound, Focused Assessment with Sonography in Trauma (FAST), and focused abdominal ultrasound examination. The treating physician was blinded to the ultrasound examination and the findings. After having performed the ultrasound examination the principal investigator conducted an interview with the treating physician. In this interview the physician was questioned to the management and workup plan for the patient. Subsequently the treating physician was unblinded to the results from the ultrasound examination. The treating physician was then again interviewed regarding patient management. At the end of this interview the physician was given five choices regarding the clinical impact of the ultrasound examination:

1. ***No new information.*** This option was considered if ultrasound revealed no pathology or pathology, which was already known by the physician. E.g. if the ultrasound examination found cholecystolithiasis, which was known from earlier hospitalization. This option was also chosen if the ultrasound was inconclusive.
2. ***New Pathology, but no further action needed.*** This option was considered if ultrasound revealed pathology, which the physician found no need to treat or investigate further. E.g. cholecystolithiasis with no relevant symptoms.
3. ***Further diagnostics needed.*** This option was considered if ultrasound examination found new pathology and the physician found it relevant to investigate further e.g. with blood test or imaging.
4. ***Presumptive diagnosis confirmed.*** This option was considered if the pathology found would confirm the suspicion, which the physician had after the physical exam. E.g. if the physician suspected pneumothorax and ultrasound examination was consistent with pneumothorax.
5. ***Immediate treatment needed*** This option was considered if new pathology was determined to be in need of immediate treatment. E.g. previously unknown large abdominal aortic aneurism in the patient with pain or unstable vital signs.

Vital signs, demographics, triage level (measured by the ED nurse), prior known disease, clinical presentation, and pathological findings were recorded on a coding sheet before the ultrasound examination was performed. All results of the ultrasound examination were recorded on a second coding sheet by the principal investigator.

### Inclusion criteria

The patient sample was a randomly selected sequence of patients over the age of 18 sampled to mimic the background population in the department. Inclusion was performed by convenience sampling when the principal investigator was present in the department and all ultrasound examinations where performed by the principal investigator. The principal investigator had no influence on which patients were included. The present study was build upon the same patients and ultrasound examinations as presented in an observational trial by Weile et al. in BMC Emergency Medicine in 2018. The random selection process is described in detail in the mentioned study [24].

The physicians included in the study where either specialist doctors, third year residents, or first year residents in the ED. All participating physicians had completed e-learning and a two-day course in POCUS which included introduction to all the examinations performed in this study. The purpose of this training was to enable the physicians to understand the ultrasound examination results.

### Sample size estimation

Based on a previous study on patients presenting with respiratory symptoms we conservatively estimated that 10% of ultrasound examinations would reveal undiscovered findings with impact on management [14]. The sample size was derived by requiring that a test on a 5% significance level *(α = 0.05)* of the hypothesis that the proportion of potentially beneficial ultrasound examinations was 8%. At completion, the study would then allow us to conclude the proportion was above 8% with a power of 80% *(β = 0.20)*, if the true proportion of potentially beneficial ultrasound examinations would be 12%. For this we would need a total of 406 patients.

### Ultrasound examinations

Focused examinations were performed of the heart, lungs, and abdomen, including examination for free fluid. The principal investigator (JW) performed all ultrasound examinations. Primary to inclusion he had performed more than 100 of each type of examination and undergone certification by three experts within lung ultrasound, cardiac ultrasound and abdominal ultrasound. An overview of all views obtained and findings, which were assessed, is provided in Table 1.

**Table 1:** Views performed in the study and predefined pathology for identification

Cardiac ultrasound	FAST	Lung ultrasound	Abdominal ultrasound
*Subxiphoid four chamber view*	*Right upper quadrant*	*Volpicelli’s eight anterolateral zones*	*Gallbladder longitudinal*
*Parasternal long axis view*	*Left upper quadrant*		*Aorta transverse view*
*Parasternal short axis view*	*Transverse view of the bladder*		*Kidneys longitudinal bilateral*
*Apical four chamber view*	*Sagittal*		*Bladder as in FAST*
*Left ventricle contractility*	*Intra peritoneal free fluid*	*Pleural effusion*	*Dilated Urinary bladder*
*Dilated right ventricle*		*Pneumothorax*	*Abdominal Aortic Aneurism*
*Pericardial fluid*		*Interstitial syndrome*	*Hydronephrosis*
		*Parenchymal pathology* *Localized multiple B-lines*	*Cholecystolithiasis*

A GE Vivid S6 (GE Healthcare, Chicago, Illinois, USA) ultrasound system was used for all ultrasound examinations. For cardiac ultrasound we used a phased array M4S-RS 1.5–3.6 MHz transducer and for all other views we used a curvilinear 4C-RS 1.8–6.0 MHz Convex Array transducer. Coded Octave Imaging and multiple-angle compound imaging features (which reduce artifacts) were turned off during focused lung ultrasound. All views were recorded as cine-loops and exported to an external hard drive in DICOM format. Post-analysis of images was performed using the ultrasound software EchoPac (GE Healthcare, Chicago, Illinois, USA).

#### Primary outcome

The primary endpoint was the proportion of patients where the results of the ultrasound examination induced changes of the physician’s plan or treatment.

#### Secondary outcomes

Secondary outcomes were changes in management according to triage level, known comorbidity, and clinical presentation. It was decided that the options “1. No new information” and “2. New pathology, but no further action needed” were aggregated into “non-beneficial POCUS examination” as these did not change the diagnostic plan or the treatment pathway. The options “3. Further diagnostics needed”, “4. Diagnosis confirmed”, and “5. Immediate treatment needed” were aggregated into “beneficial POCUS examination” because the ultrasound examination had induced a change in the patient treatment or management pathway. This dichotomization allowed logistic regression analysis.

The triage system used was the RETTS-HEV (Rapid Emergency Triage and Treatment System – HospitalsEnheden Vest) system. The system was a five-point scale with 1 (blue) as the lowest and 5 (red) as the highest. The RETTS-HEV has been validated to predict hospital length of stay and in-hospital, 30, 60, and 90-day mortality rates [25]. The known comorbidities “cardiac disease”, “hypertension”, “pulmonary disease”, “active cancer (excluding non-melanoma skin cancer)”, and “diabetes (I or II)” were chosen on the basis of known high prevalence and high mortality [26–30]. Information about comorbidity was obtained by asking the patients. The clinical presentations were based on the expected 10 most common clinical presentations found in the ED. Clinical presentations in this context refer to preliminary diagnosis or complaint displayed on an electronic dashboard in the ED. Up to two presentations could be mentioned for one patient. Minor orthopedic complaint refers to all patients presenting with complaints such as sprained knee etc. This differs from traffic accident as this presentation refers to the mechanism of injury and not the severity or complaints.

The experience of the treating physician was regarded a possible confounder and was investigated separately.

#### Statistics

Descriptive data was presented as actual numbers and percentages. Normal distributions were presented as mean with standard deviations. Non-normally distributions were presented as median with interquartile range. Normal distribution was assessed using Q-Q plots and histograms. To explore if high triage, certain prior comorbidity, and certain clinical presentation were potentially beneficial we performed a logistic regression analyses. Likewise, logistic regression was used to evaluate if specialist physicians were more reluctant to change management compared to non-specialist physicians. Results were presented as Odds Ratios with 95% confidence intervals as suggested by Andrade [31]. We used a binomial probability test to test if more than 8% of all examinations were potentially beneficial. *P*-values of less than 0.05 were considered significant. Data analysis was performed using the statistical software Stata 13 (Statacorp, Texas, USA).

## Results

**Fig. 1:**
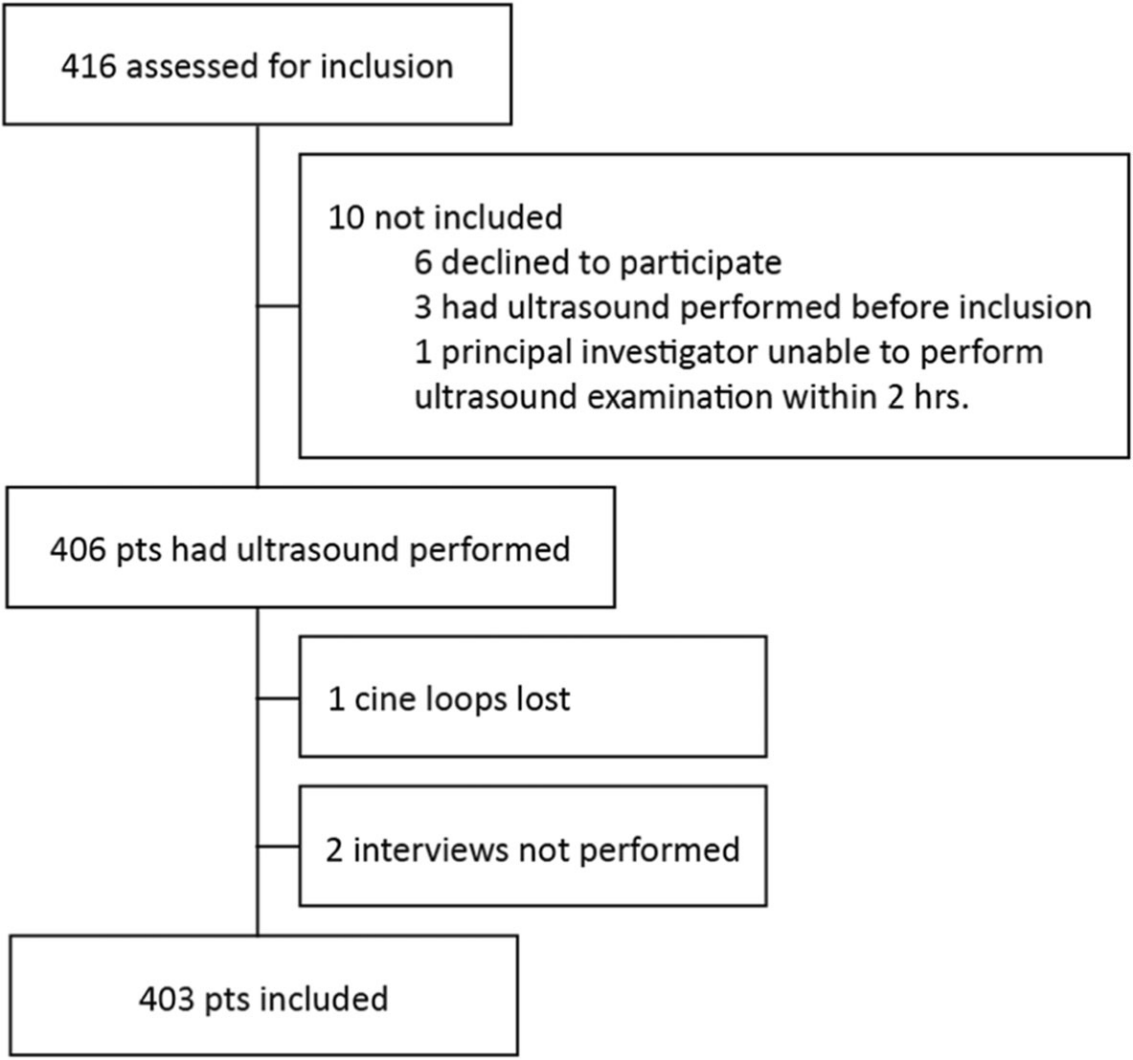
Trial profile flowchart

We screened 416 patients for eligibility between March 4th 2014 and February 23rd 2016. Ten patients were not included after primary approach. Three patients had a FAST examination performed by other physicians than the primary investigator. Six patients declined to participate, and the examination could not be performed within 2 h in one patient (Fig. 1). A total of 406 patients had the ultrasound examination performed. In one patient all cine loops were lost between recording and exporting the files from the ultrasound machine and interviews were not performed in two patients due to physician unavailability. The patient flowchart is shown in Fig. 1. At the end 403 patients with corresponding interviews were included for final analysis. The baseline characteristics are shown in Table 2 together with triage levels, known disease, and clinical presentation.

**Table 2:** Baseline characteristics including triage level, known disease and clinical presentation

Characteristic	Total (***n*** = 403)
Age, median (IQR)	55.7 (38.5; 70.1)
Male Gender, n(%)	247 (61,3)
BMI, median (IQR)	25.4 (22.9; 29.0)
BP systolic, mean (SD)	142.1 (25.6)
BP diastolic, mean (SD)	84.9 (14.9)
Temperature, median (IQR)	37.0 (36.6; 37.3)
Respiration rate, median (IQR)	16 (16; 18)
Heart rate mean (SD)	76.1 (17.5)
SpO2, median (IQR)	98 (96; 99)
Smoker n(%)	53 (13.2)
Alcohol abuse n(%)	16 (4.0)
**Triage level**	**n(%) [95% CI]**
1 (blue)	79 (19.6) [15.8; 23.8]
2 (green)	109 (27.1) [22.8; 31.7]
3 (yellow)	175 (43.4) [38.5; 48.4]
4 (orange)	34 (8.4) [5.9; 11.6]
5 (red)	6 (1.5) [0.5; 3.2]
**Known comorbidity**	**n(%) [95% CI]**
Hypertension	74 (18.4) [14.7; 22.5]
Cardiac disease	68 (16.9) [13.3; 20.9]
Pulmonary disease	40 (9.9) [7.2; 13.3]
Diabetes	32 (7.9) [5.5; 11.0]
Cancer	9 (2.2) [1.0; 4.2]
None of the above	180 (44.7) [39.7; 49.7]
**Clinical presentation**	**n (%) [95% CI]**
Orthopedic complaint	110 (27.3) [23.0; 31.9]
Abdominal pain	108 (26.8) [22.5; 31.4]
Chest pain	41 (10.2) [7.4; 13.5]
Dyspnea	40 (9.9) [7.2; 13.3]
Traffic accident	28 (6.9) [4.7; 9.9]
Unexpected fall/syncope	26 (6.5) [4.3; 9.3]
Dizziness	11 (2.7) [1.4; 4.8]
Fever	5 (1.2) [0.4; 2.9]
Chest trauma	3 (0.7) [0.2; 2.2]
Abdominal trauma	1 (0.2) [0.0; 1.4]
Other	94 (23.3) [19.3; 27.8]

*IQR* Interquartile Range, *BMI* Body Mass Index, *BP* Blood pressure, *SD* Standard Deviation, *SpO2* Peripheral oxygen saturation, *CI* Confidence interval

### Overall changes in management

**Fig. 2:**
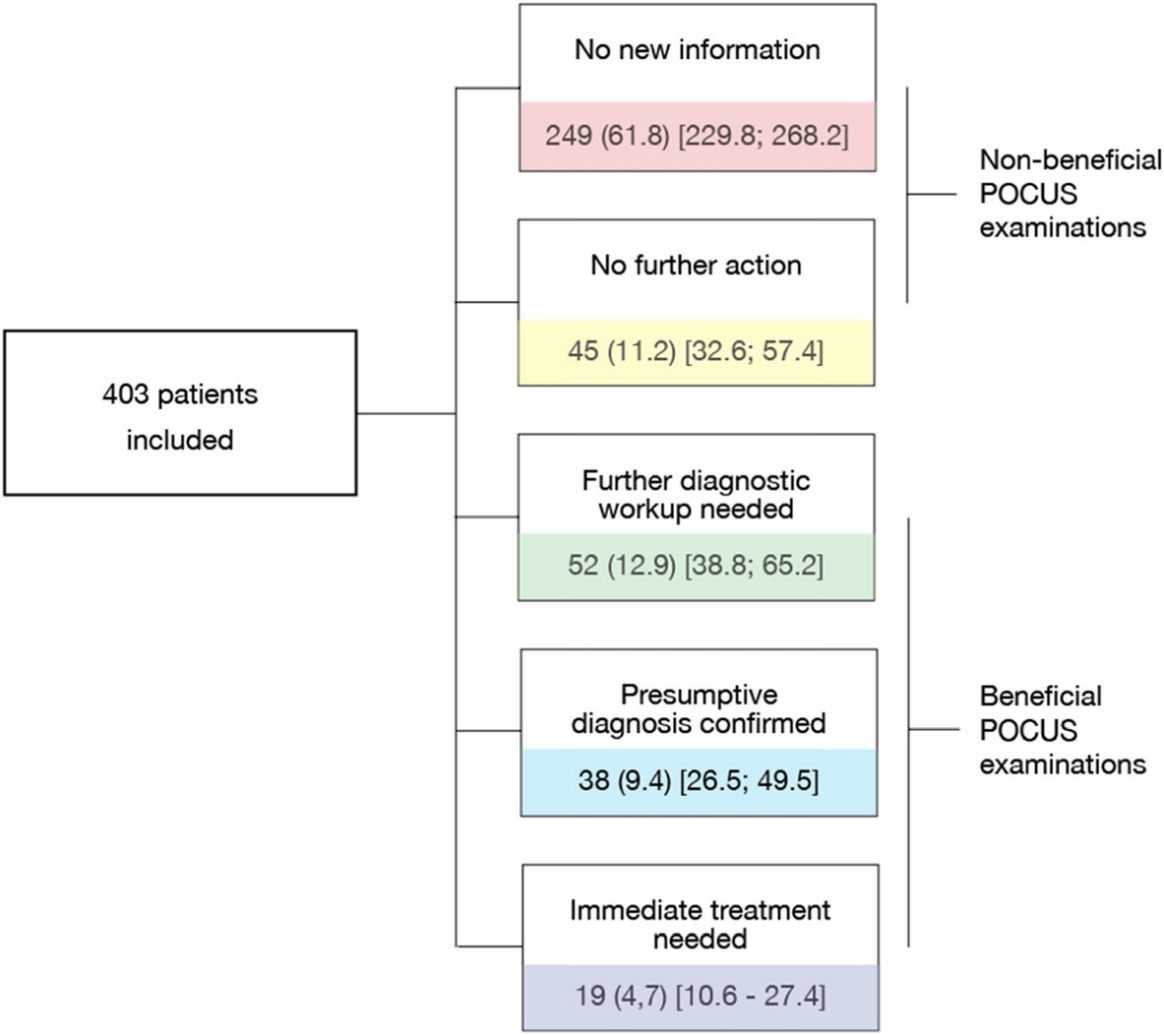
Results from the interviews with physicians when they where unblinded to the results of the ultrasound examination. The figure also illustrates which answers were considered beneficial and which where not. Results are displayed as: n(%)[95% CI]

POCUS was potentially beneficial in a total of 109 (27.0%) [95% CI: 22.7; 31.7], Significantly more than the hypothesized 8%, *p* < 0.001. Clinical management was altered due to ultrasound findings either as a choice of further diagnostic work up or immediate need of treatment in a total of 17.6% of all patients. Presumptive diagnosis was confirmed in 9.4% of all patients. The results of the interview question regarding the outcome of the ultrasound examination according to the treating physician are shown in Fig. 2. A detailed table showing the patients sorted by triage level and comorbidity and clinical presentation according to choices made by the physician regarding usefulness of the POCUS can be found as supplementary material.

### Secondary endpoints

Odds ratios of potentially beneficial ultrasound in any of the triage levels, using Triage Level 1 as reference is shown in Table 3. The table also presents odds ratio of potentially beneficial ultrasound if comorbidities were present, no comorbidity was used as reference. Furthermore the table contains a logistic regression investigating training level of the physician as a predictor for potentially beneficial POCUS with the specialist as the reference. To avoid confounding an adjusted analysis for comorbidity was also performed and the results are presented in the table.

**Table 3:** Predictors of whole body ultrasound resulting in confirmation of diagnosis, further workup or immediate treatment. OR: Odds Ratio, CI: Confidence Interval. Logistic regression of OR

Variable	Patients n	Beneficial ultrasound n (%)	Crude analysis			Adjusted analysis		
			OR	95% CI	***p***-value	OR	95% CI	***p***-value
Triage level								
1 (ref)	79	2 (2.5)	1	–	–	–	–	–
2	109	32 (29.4)	16.0	(3.7; 69.1)	< 0.001	16.1	(3.7; 71.6)	< 0.001
3	175	56 (32.0)	18.1	(4.3; 76.4)	< 0.001	17.6	(4.1; 75.8)	< 0.001
4	34	16 (47.1)	34.2	(7.2; 162.4)	< 0.001	35.4	(7.2; 173.7)	< 0.001
5	6	3 (50.0)	38.5	(4.5; 323.6)	0.001	24.1	(2.4; 243.7)	0.007
Comorbidity								
None (ref)	251	45 (17.9)	1	–	–	–	–	–
Cardiac disease	68	31 (45.6)	2.4	(1.4; 4.2)	0.003	2.3	(1.3; 4.3)	0.006
Hypertension	77	32 (41.6)	2.3	(1.3; 4.0)	0.005	2.4	(1.3; 4.4)	0.005
Pulmonary disease	40	22 (55.0)	3.5	(1.7; 7.0)	< 0.001	2.9	(1.4; 6.2)	0.004
Cancer	9	5 (55.6)	2.2	(0.5; 9.4)	0.302	3.2	(0.6; 17.3)	0.171
Diabetes	32	10 (31.3)	0.7	(0.3; 1.7)	0.479	0.6	(0.2; 1.4)	0.208
Training level								
Specialist physician (ref)	85	20 (23.5)	1	–	–			
3rd year resident	64	21 (32.8)	1.6	(0.8; 3.3)	0.211			
1st year resident	254	68 (26.7)	1.2	(0.7; 2.1)	0.555			

Table 4 shows the results of the logistic regression performed on clinical presentations. Analyses are unadjusted and adjusted for comorbidity and triage level.

**Table 4:** Logistic regression showing odds ratio for different clinical presentations as predictors of potentially beneficial ultrasound examination. CI: Confidence Interval, (*n* = 397), 5 observations left out from fever and 1 left out from abdominal trauma as these groups had no patients with no relevant ultrasound

Variable	Patients n	Beneficial ultrasound n (%)	Crude analysis			Adjusted analysis		
			OR	95% CI	***p***-value	OR	95% CI	***p***-value
Clinical presentation								
Abdominal pain	108	47 (43.5)	5.0	(2.0; 12.2)	< 0.001	5.2	(2.0; 13.4)	0.001
Chest pain	41	13 (31.7)	0.7	(0.3; 1.9)	0.554	0.9	(0.4; 2.2)	0.797
Fever	5	5 (100)	–	–	–	–	–	–
Chest trauma	3	1 (33.3)	4.0	(0.3; 50.8)	0.287	5.7	(0.4; 77.7)	0.191
Abdominal Trauma	1	0 (0)	–	–	–	–	–	–
Dyspnea	40	26 (65.0)	11.7	(4.3; 31.9)	< 0.001	10.6	(3.6; 31.2)	< 0.001
Syncope	26	9 (34.6)	2.9	(1.0; 8.4)	0.044	3.3	(1.1; 10.2)	0.038
Dizziness	11	5 (45.4)	4.8	(1.1; 20.5)	0.036	3.4	(0.7; 15.5)	0.113
Traffic accident	28	3 (10.7)	0.9	(0.2; 3.6)	0.835	0.9	(0.2; 3.9)	0.912
Minor orthopedic complaint	110	10 (9.1)	0.7	(0.3; 2.1)	0.559	1.7	(0.5; 5.5)	0.371
All Other	95	24 (25.3)	1.4	(0.7; 3.1)	0.370	1.4	(0.6; 3.3)	0.407

## Discussion

This prospective observational study of 403 unselected patients revealed statistically significant changes in management due to POCUS in one out of six patients. This was either the choice of further diagnostic workup needed or immediate change in treatment. Further, POCUS confirmed the physician’s initial diagnosis in almost one out of ten patients. In total POCUS was potentially beneficial to the physician in 27% of all cases.

The proportion of patients where POCUS was potentially beneficial in present study is surprisingly high taken into consideration that the investigated population is a broad spectrum of undifferentiated patients visiting the ED. However, in a previous study in an intensive care unit, new diagnosis was found in almost two thirds of all patients when performing POCUS [32] Two other studies performed on ICU populations also found high impact of POCUS [33, 34]. The high proportion in the present study supports the existence unexploited strengths of POCUS as amendment to the physical examination in the ED.

Secondarily the study demonstrated that triage level higher than 1 is a significant predictor of POCUS being potentially beneficial. It has been suggested that POCUS should be symptom based and focused [35]. Despite the fact that the design of the present study cannot prove causal relationship the results suggest that screening with whole-body ultrasound might be indicated if triage level is high. Further investigations are needed to validate this finding.

Clinical presentation of abdominal pain, dyspnea, or syncope also turned out to predict potentially beneficial POCUS. Finding benefit to patients with dyspnea is in alignment with previous knowledge of dyspnea being an indicator of high mortality [36] as well as previous studies showing increase in diagnostic accuracy by POCUS on patients with dyspnea [14, 37]. The findings in present study contribute further to the notion that all patients presenting in the ED with dyspnea should receive POCUS examination as an amendment to physical examination. Future studies on patients with dyspnea should focus on patient important outcomes such as mortality, length of hospitalization, risk of adverse effects, or risk of readmission. Our findings also suggest that use of POCUS is indicated on patients with abdominal pain or syncope. Syncope and abdominal pain are not as well investigated and the present trial warrants future investigations in each field. Positive results in patients with syncope were observed in only 29 patients and confirmation studies are thus warranted.

Known cardiac disease, known pulmonary disease and hypertension, but not active cancer or diabetes were predictors of beneficial POCUS. Future studies on outcomes such as mortality or hospital length of stay might support POCUS as standard of care for these patients [38].

### Limitations

The study has some limitations. First, all examinations where performed by the principal investigator. This might reduce the external validity. However, having only performed 100 of each scan, the principal investigator was not an expert when the study was initiated. This number of examinations is feasible to obtain for any emergency physician using ultrasound in his or her everyday praxis and therefor the findings are expected to be somewhat transferable.

A second limitation is the lack of follow-up on the decisions made by the emergency physicians regarding choice of change in management. Before the study all physicians received training in ultrasound, but little is known about which level of training is sufficient for physicians to interpret ultrasound findings and apply these findings into clinical context [39]. It could be speculated that insufficient knowledge would lead to over-interpretation of the ultrasound findings secondarily leading to an overuse of diagnostic testing. Previous studies have not found evidence supporting that POCUS leads to increase in unwarranted referral to further diagnostic tests [14, 40, 41]. Further research is called for to investigate the sufficient amount of experience needed to be able to apply ultrasound findings into clinical context.

Third, the indicators of clinical impact of POCUS were: further diagnostic workup needed, presumptive diagnosis confirmed, and immediate treatment needed. These were chosen because they will have potential impact on the patient treatment pathway. At least further diagnostic workup will lead to further blood samples or further imaging which might benefit the patient. Immediate treatment might be lifesaving in some cases. We chose to make a conglomerate of all three outcomes, which would potentially benefit the patient, as a binary outcome was necessary for the logistic regression. If not sorted into binary groups more patients would have been needed for such a study because small groups were vulnerable to chance. Hence, the present study only served to identify subgroups for further investigation.

Fourth, it could be speculated that the physicians would be biased to respond on the ultrasound results due to Hawthorne Effect [42]. The design of the present study does not allow blinding the physicians to the fact that a study is being undertaken and hence, the risk of Hawthorne Effect is complex to avoid. The design does, however, allow the physician to be blinded to the ultrasound results while being interviewed regarding the initial plan. Previous studies on benefit of ultrasound are based on physician self reporting [43]. When the physicians report the effect of the ultrasound examination it is prone to cognitive choice-supportive bias [44] rendering the importance of ultrasound higher than it really is. Yet not perfect, the present blinded design is stronger than self-reporting regarding the sole effect of the ultrasound as an amendment to the physical examination.

Fifth, the study was a single center study in a Danish ED, which only received referred patients. Hence, the group of patients included in this study might be more severely ill in general if compared to an emergency facility with patients without referral. The influence of POCUS on the unselected patients with illness of lower severity might be lower than in the present study.

## Conclusion

POCUS as amendment to the physical examination in unselected ED patients revealed findings beneficial for clinical management in one out of six patients. Further we found that high triage score, known cardiac disease, hypertension, pulmonary diseases, a clinical presentation with abdominal pain, dyspnea or syncope are predictors of POCUS being beneficial. Future research should focus on the clinical effect of POCUS on patient-important outcomes.

## Supplementary information

**Additional file 1: Table S1.** Triage level and comorbidities distribution according to category chosen by the treating physician. HTN: Hypertension. All number are presented as n (%) [95% CI].

## Data Availability

The data that support the findings of this study are available from the corresponding author upon reasonable request.

## Abbreviations

POCUS: Point-of-Care Ultrasound
ED: Emergency Department
FAST: Focused Assessment with Sonography in Trauma
