# Supplementary material for: Point-of-care ultrasound induced changes in management of unselected patients in the emergency department - a prospective single-blinded observational trial
Source: Scand J Trauma Resusc Emerg Med. 2020 May 29;28:47. doi: 10.1186/s13049-020-00740-x (PMC7260768; doi:10.1186/s13049-020-00740-x)
Supplement: Supplementary file 1 — Additional file 1: Table S1. Triage level and comorbidities distribution according to category chosen by the treating physician. HTN: Hypertension. All number are presented as n (%) [95% CI]. [file 13049_2020_740_MOESM1_ESM.docx]

***Table 3:*** *Triage level and comorbidities distribution according to category chosen by the treating physician. HTN: Hypertension. All number are presented as n (%) [95% CI]*

|  | | **Triage level** | |  | | |  | | | |  | | | | |  | | | **Comorbidities** | | |  | |  | |  | |  | |  |
| --- | --- | --- | --- | --- | --- | --- | --- | --- | --- | --- | --- | --- | --- | --- | --- | --- | --- | --- | --- | --- | --- | --- | --- | --- | --- | --- | --- | --- | --- | --- |
|  | | **1** | | **2** | | | **3** | | | | **4** | | | | | **5** | | | **HTN** | | | **cardiac** | | **pulmonary** | | **cancer** | | **diabetes** | |  |
| ***Non-beneficial POCUS*** | |  | |  | | |  | | | |  | | | | |  | | |  | | |  | |  | |  | |  | |  |
| 1. No new information | | 68 (86.1) [76.5; 92.8] | | 62 (56.9) [47.0; 66.3] | | | 103 (58.9) [51.2; 66.2] | | | | 16 (47.1) [29.8; 64.9] | | | | | 0 (0) [0.0; 45.9] | | | 35 (45.5) [34.1; 57.2] | | | 25 (36.8) [24.4; 49.3] | | 11 (27.5) [14.6; 43.9] | | 2 (22.2) [2.8; 60.0] | | 20 (62.5) [43.7; 78.9] | |  |
| 2. New Pathology, but no further action needed | | 9 (11.4) [5.3; 20.5] | | 15 (13.8) [7.9; 21.7] | | | 16 (9.1) [5.3; 14.4] | | | | 2 (5.9) [0.7; 19.7] | | | | | 3 (50.0) [11.8; 88.2] | | | 7 (9.1) [3.7; 17.8] | | | 12 (17.6) [9.5; 28.8] | | 7 (17.5) [7.3; 32.8] | | 2 (22.2) [2.8; 60.0] | | 2 (6.3) [0.8; 20.8] | |  |
| *Sub Total* | | *77 (97.5) [91.1; 99.7]* | | *77 (70.6) [61.2; 79.0]* | | | *119 (68.0) [60.5; 74.8]* | | | | *18 (52.9) [35.1; 70.2]* | | | | | *3 (50.0) [11.8; 88.2]* | | | *45 (58.4) [46.7; 69.6]* | | | *37 (54.4) [30.4; 53.4]* | | *18 (45.0) [29.3; 61.5]* | | *4 (44.4) [13.7; 78.8]* | | *22 (68.8) [50.0; 83.9]* | |  |
|  | |  | |  | | |  | | | |  | | | | |  | | |  | | |  | |  | |  | |  | |  |
| ***Potentially beneficial POCUS*** | |  | |  | | |  | | | |  | | | | |  | | |  | | |  | |  | |  | |  | |  |
| 3. Further diagnostic workup needed | | 1 (1.3) [0.0; 6.8] | | 20 (18.3) [11.5; 26.9] | | | 26 (14.9) [9.9; 21.0] | | | | 4 (11.8) [3.3; 27.4] | | | | | 1 (16.7) [0.4; 64.1] | | | 16 (20.8) [12.4; 31.5] | | | 17 (25.0) [15.3; 37.0] | | 10 (25) [12.7; 41.2] | | 2 (22.2) [2.8; 60.0] | | 3 (9.4) [2.0; 25.0] | |  |
| 4. Presumptive diagnosis confirmed | | 0 (0) [0.0; 4.6] | | 7 (6.4) (2.6; 12.8) | | | 21 (12.0) [7.6; 17.8] | | | | 8 (23.5) [10.7; 41.1] | | | | | 2 (33.3) [4.3; 77.7] | | | 11 (14.3) [7.4; 24.1] | | | 9 (13.2) [6.2; 23.6] | | 8 (20.0) [9.1; 35.6] | | 2 (22.2) [2.8; 60.0] | | 5 (15.6) [5.3; 32.8] | |  |
| 5. Immediate treatment needed | | 1 (1.3) [0.0; 6.8] | | 5 (4.6) [15.1; 10.4] | | | 9 (5.1) [2.4; 9.5] | | | | 4 (11.8) [3.3; 27.4] | | | | | 4 (66.7) [22.3; 95.7] | | | 5 (2.8) [2.1; 14.5] | | | 5 (7.4) [2.4; 16.3] | | 4 (10.0) [2.8; 23.7] | | 1 (11.1) [0.2; 48.2] | | 2 (6.3) [0.8; 20.8] | |  |
| *Sub Total* | | *2 (2.5) [0.3; 8.8]* | | *32 (29.4) [21.0; 38.8]* | | | *56 (32.0) [25.2; 39.5]* | | | | *16 (47.1) [29.8; 64.9]* | | | | | *3 (50.0) [11.8; 88.2]* | | | *32 (41.6) [30.4; 53.4]* | | | *31 (45.6) [33.5; 58.1]* | | *22 (55.0) [38.5; 70.7]* | | *5 (55.6) [21.2; 86.3]* | | *10 (31.1) [16.1; 50.0]* | |  |
|  | |  | |  | | |  | | | |  | | | | |  | | |  | | |  | |  | |  | |  | |  |
| **Total** | | **79** | | **109** | | | **175** | | | | **34** | | | | | **6** | | | **77** | | | **68** | | **40** | | **9** | | **32** | |  |
|  |  | | | |  | | |  | |  | |  |  | |  | | |  | |  |  | | | | | | | | | |
|  | **Clinical presentation** | | | |  | | |  | |  | |  |  | |  | | |  | |  |  | | | | | | | | | |
|  | **Abdominal pain** | | **Chest Pain** | | | **Fever** | | | **Chest trauma** | | | | | **Abd trauma** | | | **Dyspnea** | | | **Syncope** | | | **Dizziness** | | **Traffic accident** | | **Minor orthopedic complaint** | | **All Other** | |
| ***Non-beneficial POCUS*** |  | |  | | |  | | |  | | | | |  | | |  | | |  | | |  | |  | |  | |  | |
| 1. No new information | 48 (44.4) [34.9; 54.3] | | 25 (61.0) [44.5; 75.8] | | | 0 (0) [0.0; 52.2] | | | 2 (66.7) [9.4; 99.2] | | | | | 1 (100) [2.5; 100] | | | 13 (32.5) [18.6; 49.1] | | | 15 (57.7) [36.9; 76.6] | | | 5 (45.4) [16.7; 76.6] | | 21 (75.0) [55.1; 89.3] | | 84 (76.4) [67.3; 83.9] | | 61 (64.2) [53.7; 73.8] | |
| 2. New Pathology, but no further action needed | 13 (12.0) [6.6; 19.7] | | 3 (7.3) [1.5; 19.9] | | | 0 (0) [0.0; 52.2] | | | 0 (0) [0.0; 70.7] | | | | | 0 (0) [0.0; 97.5] | | | 1 (2.5) [0.1; 13.1] | | | 2 (7.7) [0.9; 25.1] | | | 1 (9.0) [0.2; 41.3] | | 4 (14.2) [4.0; 32.7] | | 16 (14.5) [8.5; 22.5] | | 10 (10.5) [5.2; 18.5] | |
| *Sub Total* | *61 (56.5) [46.6; 66.0]* | | *28 (68.3) [51.9; 81.9]* | | | *0 (0) [0.0; 52.2]* | | | *2 (66.7) [9.4; 99.2]* | | | | | *1 (100) [2.5; 100]* | | | *14 (35.0) [20.6; 51.7]* | | | *17 (65.4) [44.3; 82.8]* | | | *6 (54.5) [23.4; 83.3]* | | *25 (89.3) [71.8; 97.7]* | | *100 (90.9)[83.9; 95.6]* | | *71 (74.7) [64.8; 83.1]* | |
|  |  | |  | | |  | | |  | | | | |  | | |  | | |  | | |  | |  | |  | |  | |
| ***Potentially beneficial POCUS*** |  | |  | | |  | | |  | | | | |  | | |  | | |  | | |  | |  | |  | |  | |
| 3. Further diagnostic workup needed | 23 (3.9) [14.0; 30.2] | | 6 (14.6) [5.6; 29.2] | | | 2 (40.0) [5.3; 85.3] | | | 0 (0) [0.0; 70.7] | | | | | 0 (0) [0.0; 97.5] | | | 10 (25) [12.7; 41.2] | | | 7 (26.9) [11.6; 47.8] | | | 4 (36.4) [10.9; 69.2] | | 1 (3.6) [0.1; 18.3] | | 4 (3.6) [1.0; 9.0] | | 11 (11.6) [5.9; 19.8] | |
| 4. Presumptive diagnosis confirmed | 19 (17.6) [10.9; 26.1] | | 7 (17.1) [7.2; 32.1] | | | 3 (60.0) [14.7; 94.7] | | | 0 (0) [0.0; 70.7] | | | | | 0 (0) [0.0; 97.5] | | | 10 (25) [12.7; 41.2] | | | 2 (7.7) [0.9; 25.1] | | | 1 (9.0) [0.2; 41.3] | | 0 (0) [0.0; 12.3] | | 3 (2.7) [0.6; 7.8] | | 9 (9.5) [17.2; 4.4] | |
| 5. Immediate treatment needed | 5 (4.6) [1.5; 10.5] | | 0 (0) [0.0; 8.6] | | | 0 (0) [0.0; 52.2] | | | 1 (33.3) [0.8; 90.6] | | | | | 0 (0) [0.0; 97.5] | | | 6 (15.0) [5.7; 29.8] | | | 0 (0) [0.0; 13.2] | | | 0 (0) [0.0; 28.5] | | 2 (7.1) [0.9; 23.5] | | 3 (2.7) [0.6; 7.8] | | 4 (4.2) [1.2; 10.4] | |
| *Sub Total* | *47 (43.5) [34.0; 53.4]* | | *13 (31.7) [18.1; 48.1]* | | | *5 (100) [47.8; 100]* | | | *1 (33.3) [0.8; 90.6]* | | | | | *0 (0) [0.0; 97.5]* | | | *26 (65.0) [48.3; 79.4]* | | | *9 (34.6) [17.2; 55.7]* | | | *5 (45.4) [16.7; 76.6]* | | *3 (10.7) [2.3; 28.2]* | | *10 (9.1) [4.4; 16.1]* | | *24 (25.3) [16.9; 35.2]* | |
|  |  | |  | | |  | | |  | | | | |  | | |  | | |  | | |  | |  | |  | |  | |
| **Total** | **108** | | **41** | | | **5** | | | **3** | | | | | **1** | | | **40** | | | **26** | | | **11** | | **28** | | **110** | | **95** | |
